# c-Myb-mediated inhibition of miR-601 in facilitating malignance of osteosarcoma via augmentation of PKMYT1

**DOI:** 10.1038/s41598-022-10684-0

**Published:** 2022-04-23

**Authors:** Peng Luo, Jiarui Fang, Houqing Chen, Feng He, Siying Xiao, He Liu, Shizhuang Zhu, Jianzhou Luo, Changqing Jiang

**Affiliations:** Department of Orthopedics, Huazhong University of Science and Technology Union Shenzhen Hospital, Shenzhen, 518000 China

**Keywords:** Cancer, Medical research, Oncology

## Abstract

The crosstalk between osteosarcoma (OS) development and abnormally expressed microRNA (miR)-601 is not explored explicitly. Here, we identified the downregulated miR-601 in osteosarcoma (OS) through a comprehensive bioinformatics analysis of GEO Datasets. The results indicated that miR-601 was downregulated in both OS cells and tissues. The OS patients with reduced expression of miR-601 displayed worse prognosis. The results of in vitro and in vivo assay revealed that elevated miR-601 inhibited the proliferative, migratory and invasive capacities in OS cells. Mechanically, miR-601 exerted its function via targeting oncogene protein kinase membrane associated tyrosine/threonine 1 (PKMYT1) at post-transcriptional level. Moreover, miR-601 was attenuated by c-Myb at transcriptional level. Taken together, our studies reveal that miR-601 is a suppressive gene negatively correlated with malignancy of OS.

## Introduction

Osteosarcoma (OS), the most common osteogenic malignant tumor, is most commonly seen in children and adolescents^1,2^. It is a heterogeneous tumor with complex pathogenesis, and the pathogenesis has not been fully clarified. Although the incidence of OS is not high, it has a high mortality rate of disability, which brings great mental and economic burden to patients, their families and society^3^. Pulmonary metastasis is a common cause of death in OS patients. Although surgical resection combined with neoadjuvant chemotherapy reduced mortality in OS patients, 5 year survival remained low, approximately 70% in non-metastatic patients compared with 11 to 30% in metastatic patients^4^. Despite numerous progress has been made, the commitments for early detection, novel therapies and lower mortality are largely futile.

The protein kinase membrane associated tyrosine/threonine 1 (PKMYT1) gene encodes a member of the serine/threonine Protein kinase family^5,6^. The protein of PKMYT1 is a membrane-associated kinase, which regulates cell cycle negatively via phosphorylating and extinguishing cyclin-dependent kinase 1 during G2/M phase^7^. Recent studies have shown that PKMYT1 is overexpressed in many malignant tumors^8,9^. Meanwhile, PKMYT1 has been reported to drive the progression of colorectal cancer^9^, prostate cancer^10^, gastric cancer^11^, non-small cell lung cancer^12^. A literature has reported that PKMYT1 is upregulated in OS tissues^13^. And high levels of PKMYT1 had lower rates of both overall survival and metastasis-free survival of OS patients than those with low levels of PKMYT1^13^. However, the underlying mechanisms of PKMYT1 in OS remains dimly.

Recently, microRNAs (miRNAs) have been illustrated to play crucial roles in various physiological conditions, as well as pathological conditions, including OS^14,15^. Owing to the role in the progression, invasion and metastasis of OS cells, microRNA has been validated as a certain potential as a prognostic indicator or therapeutic target. Despite lots of miRNAs have been validated as promoters or suppressors in the generation and progression of OS, such as miR-140^16^, miR-328-3p^17^, miR-34a^18^, miR-21^19^ and miR-215-5p^20^, miR-601 has not been systematically researched in OS.

Here, we aimed to elucidate the specific roles miR-601 involved in OS. The microarray results indicated that miR-601 was lowly expressed in OS cells. Our results showed the same trend in OS cells and tissues. Furthermore, attenuated level of miR-601 in OS patients showed a poor prognosis. Additionally, we found that miR-601 inhibited OS progression by targeting PKMYT1. We also clarified that miR-601 was repressed by the upstream transcription factor of c-Myb.

## Results

### miR-601 is downregulated in OS and related to poor outcome

**Figure 1:**
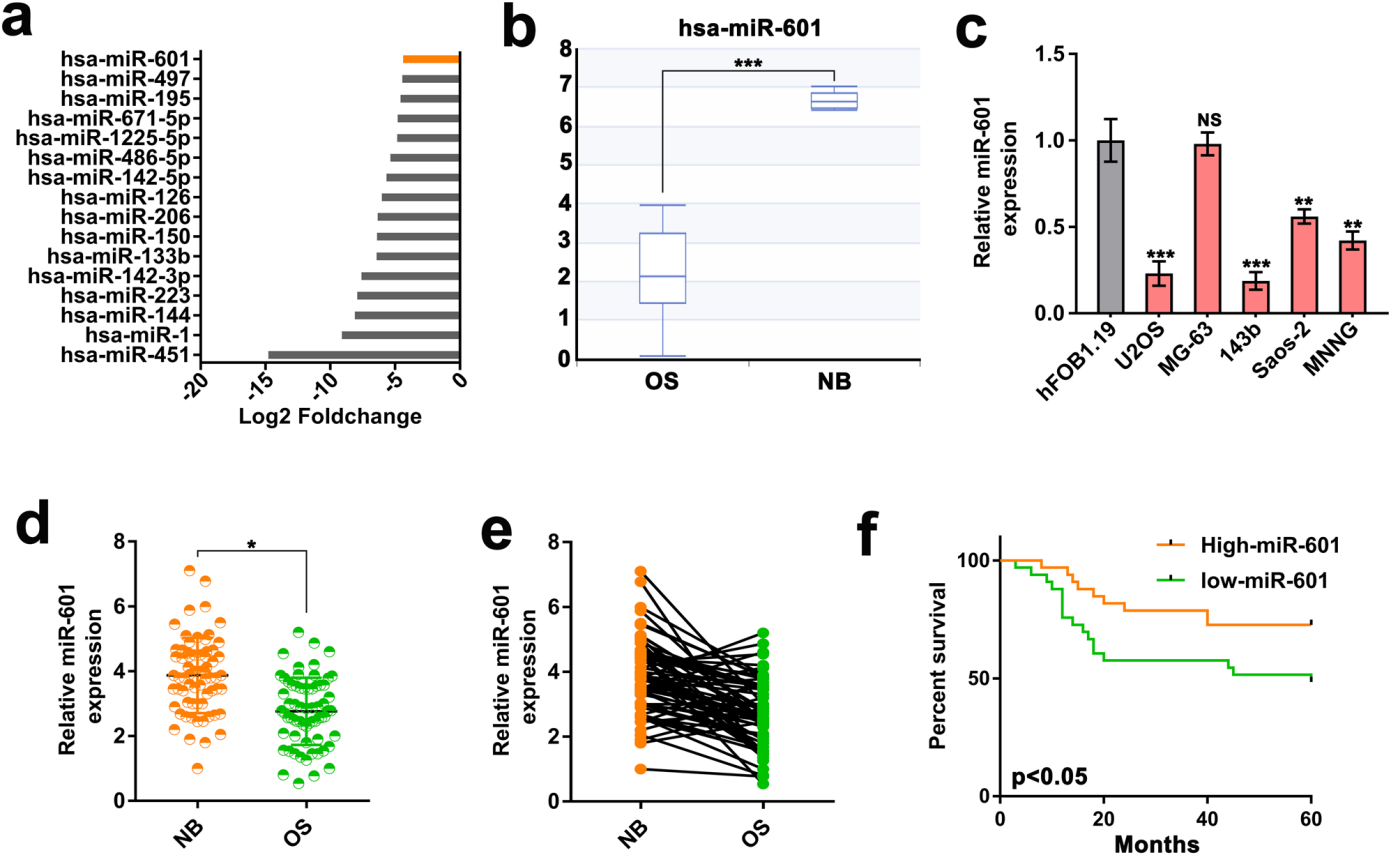
miR-601 is downregulated in OS and related to poor outcome. (**a**) A histogram showed the 16 most downregulated miRNAs in OS cells compared with normal osteocytes. (**b**) The histogram showed the miR-601 expression in OS cells compared with normal osteocytes. (**c**) The miR-601 expression was evaluated by RT-qPCR assay in 5 OS cell lines and normal bone cells. (**d, e**) The expression of miR-601 was evaluated by RT-qPCR assay in 66 paired OS tissues and normal tissues (NB). (**f**) Kaplan–Meier curves analysis showed overall survival of 66 OS patients in miR-601 high and low groups. Patients were divided into "high" and "low" group with the 50^th^ percentile expression of miR-601 as the critical point.

To identify the functional miRNA in OS, we conducted a bioinformatics analysis via utilizing the miRNA expression profiles of GSE: 28,423 in GEO datasets. Among the differentially expressed miRNAs in 4 normal bone cell lines and 19 OS cell lines, we found that the role and mechanism of down-regulated miR-601 in OS are rarely reported (Fig. 1a). The results of differential analysis showed that miR-601 was dramatically down-regulated in OS cells (Fig. 1b). Consistently, RT-qPCR results further supported that miR-601 was dramatically reduced in 4 OS cells lines compared with the normal bone cells (hFOB1.19) (Fig. 1c). Meanwhile, we further measured the level of miR-601 in 66 tissues and paired OS normal tissues using RT-qPCR analysis. The results suggested that miR-601 was remarkably reduced in OS tissues compared with adjacent normal tissues (Fig. 1d, e). Moreover, the association between the overall survival of OS patients and miR-601 expression was verified by Kaplan–Meier analysis. The result suggested that lower miR-601 level was associated with shorter overall survival (Fig. 1f). Based on the aforementioned information, we concluded that miR-601 was downregulated in OS, and that lower miR-601 expression was correlated with poor prognosis in OS.

### Overexpression of miR-601 promotes proliferation and metastasis of OS cells

**Figure 2:**
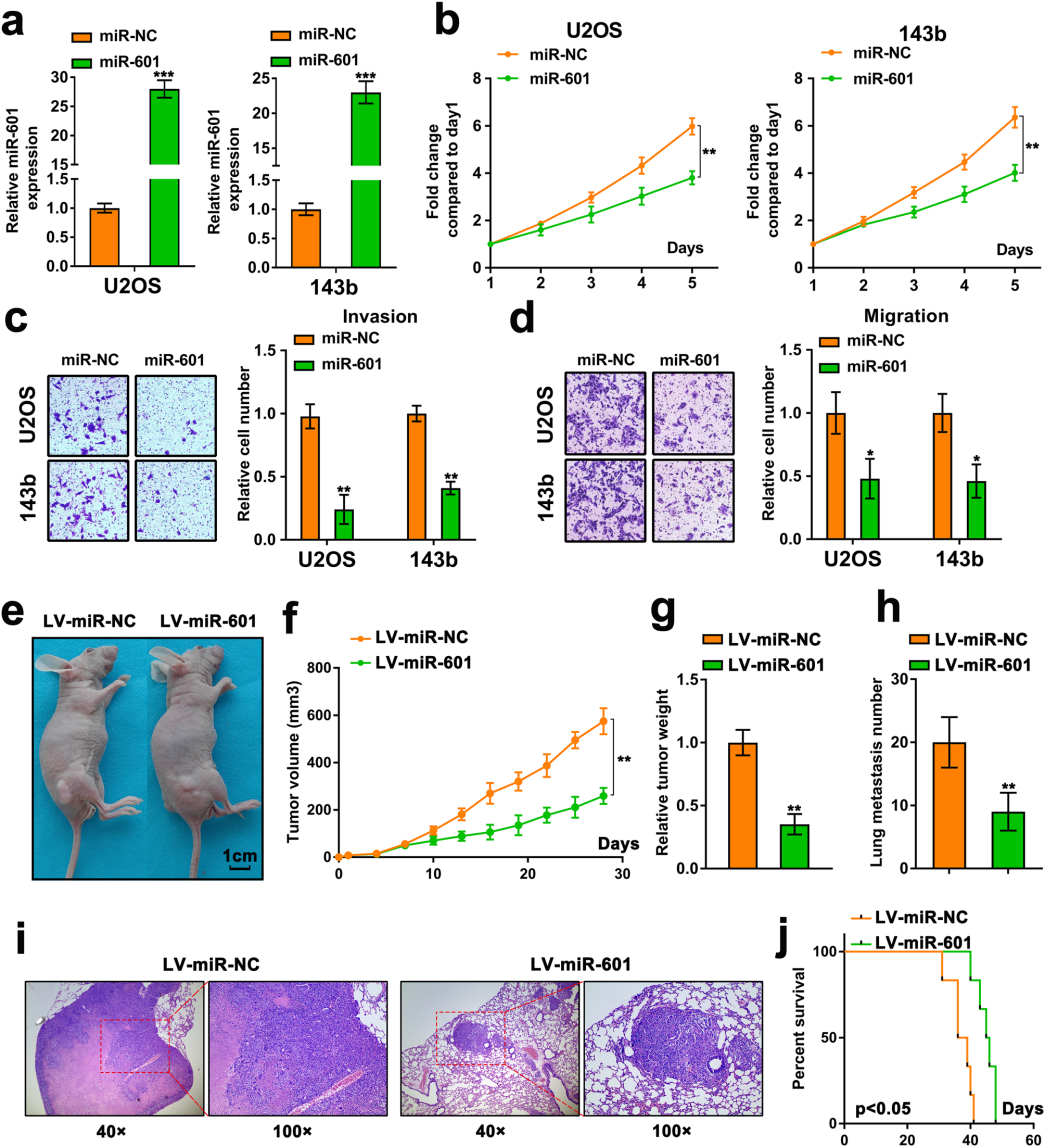
Overexpression of miR-601 promotes proliferation and metastasis of OS cells. (**a**) The miR-601 expression was evaluated by RT-qPCR assay in U2OS and 143b cells with miR-601 overexpression. (**b**) The proliferation of U2OS/143b cells was measures by the MTT experiment. (**c, d**) The invasion and migration abilities of U2OS and 143b cells was assessed by the transwell assay. (**e**) The nude mice (6 per group) were subcutaneously injected with U2OS cells transfected with lentivirus vector carrying the miR-601 sequence (LV-miR-601) or negative control (LV-miR-NC). (**f, g**) The volume and weight of xenograft tumor of nude mice. (**h, i**) U2OS cells, transfected with lentivirus vector carrying the miR-601 sequence (LV-miR-601) or negative control (LV-miR-NC), were injected into the nude mice (6 per group) from tail vein. The metastasis on lung was assessed via H&E staining. (**j**) The survival of nude mice was analyzed.

To elucidate the biological function of miR-601 in OS cells, miR-601 mimic were used to upregulate the expression of miR-601, and the efficiency was verified via RT-qPCR assay (Fig. 2a). The MTT assay results indicated that the proliferation abilities was dramatically decreased in OS cells with miR-601 overexpression (Fig. 2b). Meanwhile, the transwell assay results suggested that miR-601 upregulation remarkably impaired the migration and invasion of OS cells (Fig. 2c, d). Moreover, subcutaneous xenograft model and pulmonary metastasis model were performed to confirm the biological function of miR-601 in vivo. In accord with the in vitro results, miR-601 overexpression remarkably reduced the tumor volume and weight compared with the control group (Fig. 2e-g). Furthermore, mice injected with OS cells, transfected with LV-miR-601, showed decreased number of metastatic foci in their lungs compared with those in the mice of control group (Fig. 2h). The results of hematoxylin and eosin (H&E) staining indicated that miR-601 overexpression dramatically reduced the volume of lung metastatic lesions compared with the control group (Fig. 2i). What’s more, the mice in the miR-601 overexpression group showed higher survival rate than the control group (Fig. 2j). Collectively, the results suggested that miR-601 repressed the proliferation and metastasis of OS cells.

### miR-601 mediates the downregulation of PKMYT1

**Figure 3:**
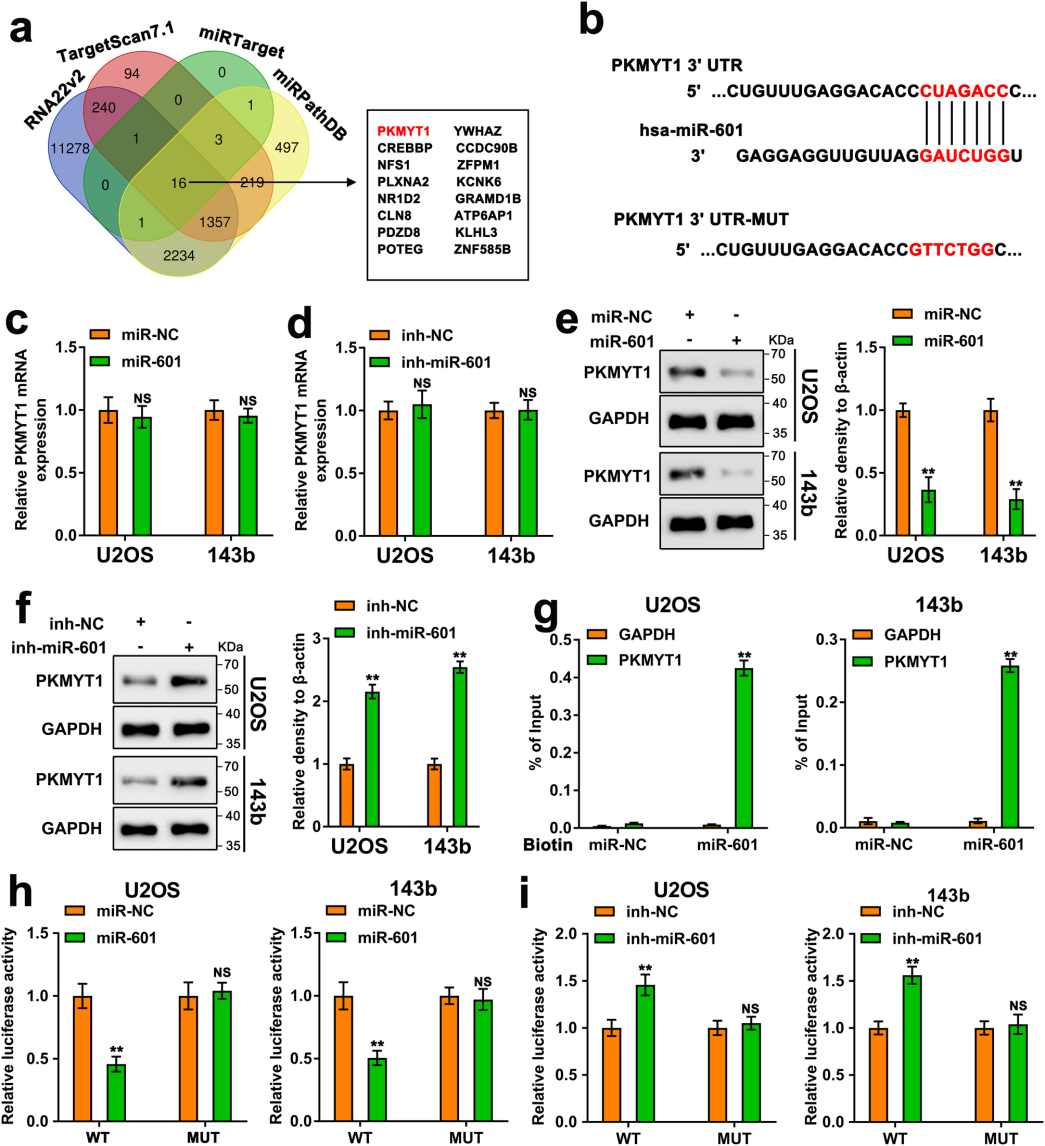
miR-601 mediates the downregulation of PKMYT1. (**a**) Venn diagram displayed the targets of miR-601 predicted by the databases of RNA22v2, TargetScam 7.1, miRTarget and miRPathDB. (**b**) Schematic diagram displayed the putative binding sequence of 3’-UTR-PKMYT1- mRNA and miR-601. The mutated sites of the 3’-UTR-PKMYT1- mRNA was showed. (**c, d**) The expression of PKMYT1 mRNA in miR-601 upregulated or downregulated U2OS and 143b cells was measured via RT-qPCR experiment. (**e, f**) The protein of PKMYT1 in miR-601 upregulated or downregulated U2OS and 143b cells was measured via Western blot experiment. The original blots/gels are presented in Supplementary Dataset File. (**g**) The enrichment of PKMYT1 on biotinylated miR-NC and miR-601 probe was evaluated by biotin-based pulldown assay. The GAPDH mRNA served as control. (**h, i**) Luciferase activity of U2OS and 143b cells was accessed through luciferase reporter assay.

To validate the potential target of miR-601, the databases of RNA22v2, TargetScan7.1, miRTarget and miRPathDB were utilized (Fig. 3a). Among the 16 genes screened from the above databases, PKMYT1 was reported to be implicated in various cancer types ^6^. Meanwhile, the putative miR-601 binding sites were predicted in PKMYT1 (Fig. 3b). The RT-qPCR results showed that both miR-601 overexpression and miR-601 depletion could not affect the mRNA level of PKMYT1 (Fig. 3c, d). Whereas, miR-601 overexpression remarkably reduced the protein level of PKMYT1 (Fig. 3e). In addition, depletion of miR-601 had the opposite effect (Fig. 3f), indicating that miR-601 regulated the expression of PKMYT1 in posttranscriptional level. Next, we ought to prove the direct interaction between miR-601 and PKMYT1 mRNA. The biotin based RNA pulldown assay indicated that there was a direct combination between miR-601 and the mRNA of PKMYT1 (Fig. 3g). Furthermore, in order to demonstrate the specificity of binding sites, Luciferase reporting experiments were performed. The wild-type and mutant PKMYT1-mRNA-3 'UTR sequences were inserted into the luciferase reporter plasmids to construct the luciferase reporter plasmids of PKMYT1-3' UTR (WT: wild-type; MUT: mutant-type). The results suggested that overexpression of miR-601 reduced the WT luciferase activity, while inhibition of miR-601 enhanced the WT luciferase activity (Fig. 3h, i). However, the change of miR-601 expression had no significant effect on the luciferase activity of the MUT. These results suggest that miR-601 regulated PKMYT1 translation via binding to the region of PKMYT1-mRNA-3'UTR. Taken together, PKMYT1 is regulated by miR-601 at posttranscriptional level and a potential target of miR-601.

### miR-601 exerts its tumor suppressor function in OS by targeting PKMYT1

**Figure 4:**
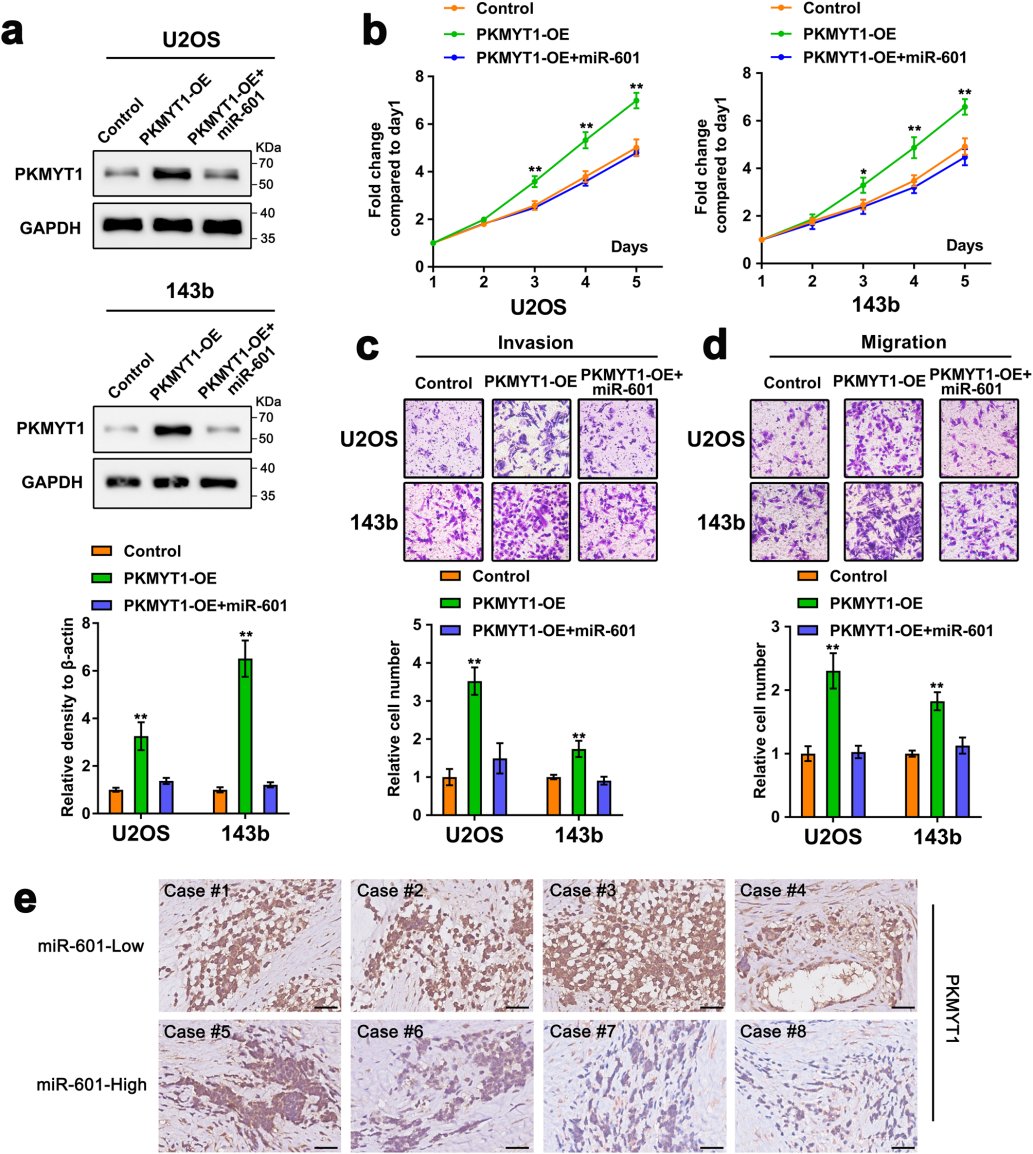
miR-601 exerts its tumor suppressor function in OS by targeting PKMYT1. (**a**–**d**) U2OS/143b cells were transfected with PKMYT1 overexpression plasmid, miR-601 mimic or negative control. (**a**) The protein level of PKMYT1 were detected by Western blot assay in U2OS and 143b cells. The original blots/gels are presented in Supplementary Dataset File. (**b**–**d**) MTT and transwell assays assess the proliferation, invasion and migration of U2OS and 143b cells. (**e**) IHC assay detected the PKMYT1 protein expression in OS tissues with miR-601-low and miR-601-high. Scale bar: 100 μm.

Next, we continued to explore that miR-601 played its function of inhibiting proliferation and metastasis through regulating the level of its downstream target PKMYT1 in OS. The results indicated that upregulation of miR-601 significantly inhibited the protein level of endogenous PKMYT1 (Fig. 4a). MTT assay suggested that upregulation of PKMYT1 significantly facilitated the proliferation of OS cells, which was neutralized by overexpression of miR-601 (Fig. 4b). Similarly, it was observed that elevated expression of miR-601 dramatically attenuated the enhanced invasion and metastasis induced by augmentation of PKMYT1 (Fig. 4c, d). Moreover, we also detected the PKMYT1 protein level in OS tissues via Immunohistochemistry (IHC) assay. The results revealed that PKMYT1 expression was high in OS tissues with low miR-601-expression and low in OS tissues with high miR-601-expression (Fig. 4e), suggesting a negative correlation between miR-601 expression and PKMYT1 protein expression in OS tissues. These results above revealed that PKMYT1 functioned as a tumor-promotor in OS cells, and miR-601 exerted a tumor suppressor effect by inhibiting its expression.

### c-Myb is involved in the downregulation of miR-601 in OS cells

**Figure 5:**
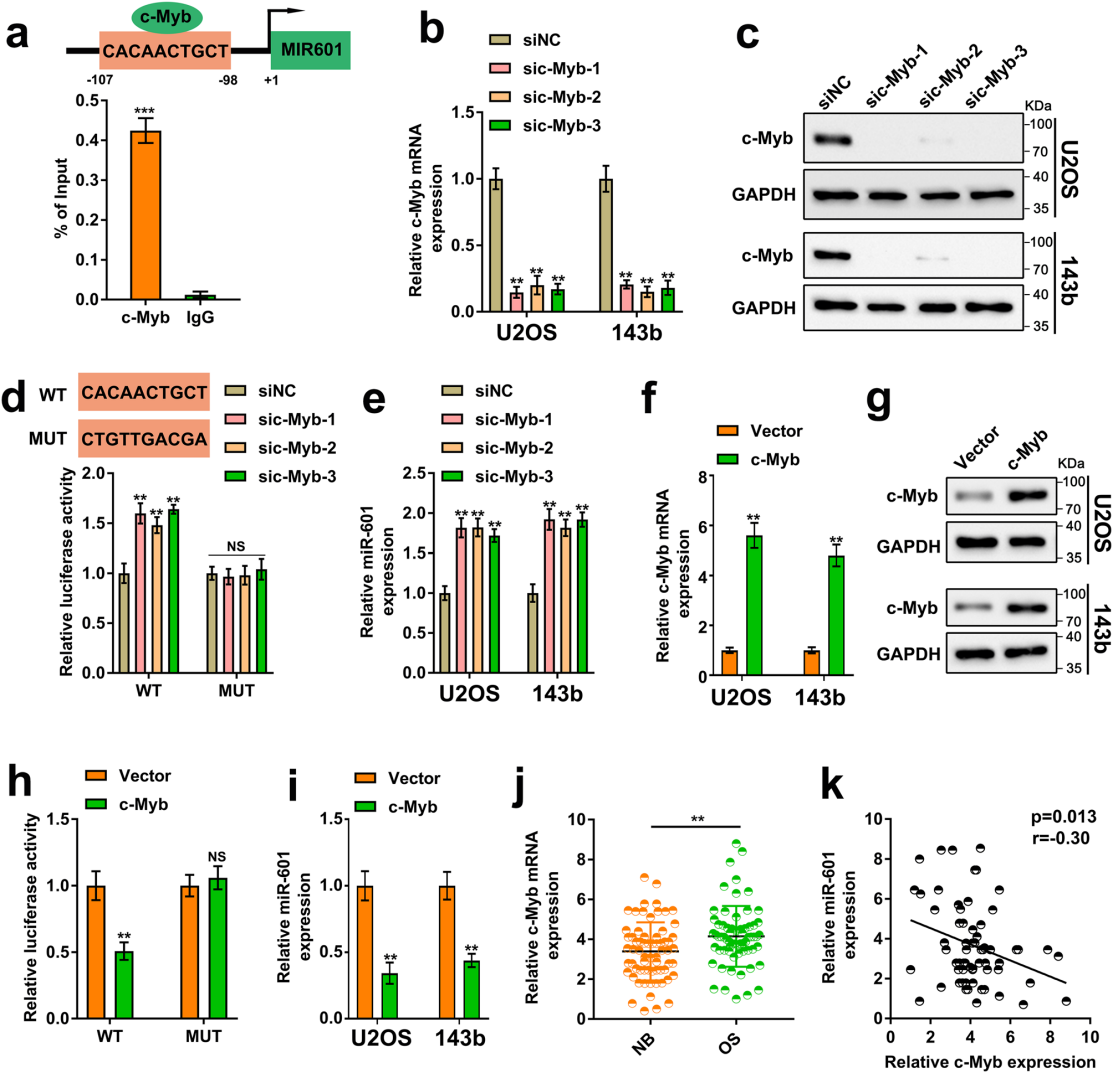
c-Myb is involved in the downregulation of miR-601 in OS cells. (**a**) (Up) Schematic diagram displayed the putative c-Myb binding sites on the promoter of MIR601. (Down) Chip assay was performed using anti-c-Myb or anti-IgG antibodies. (**b, c**) The knockdown efficiency of c-Myb was evaluated by RT-qPCR assay and Western blot assay. The original blots/gels are presented in Supplementary Dataset File. (**d**) (Up) Schematic diagram displayed the wild type and mutant type of putative binding sites of c-Myb on the promoter of MIR601. (Down) The luciferase activity of c-Myb knockdown U2OS and 143b cells, co-transfected with luciferase reporter plasmid coding MIR601 promoter sequence or scrambled vector, was accessed by luciferase reporter assay. (**e**) The miR-601 expression was evaluated by RT-qPCR assay in U2OS and 143b cells with c-Myb knockdown. (**f, g**) The level of c-Myb mRNA and protein were detected via RT-qPCR and Western blot assay in c-Myb overexpression U2OS and 143b cells. The original blots/gels are presented in Supplementary Dataset File. (**h**) The luciferase activity of c-Myb overexpression U2OS and 143b cells, co-transfected with luciferase reporter plasmid coding MIR601 promoter sequence or scrambled vector, was accessed by luciferase reporter assay. (**i**) The miR-601 expression was evaluated by RT-qPCR assay in U2OS and 143b cells. (**j**) The mRNA level of c-Myb were measured via RT-qPCR assay in 66 paired OS tissues and normal tissues. (**k**) Pearson correlation analysis displayed the correlation between miR-601 and c-Myb in OS tissues.

To explore the underlying mechanism of miR-601 downregulation in OS, the transcription factors that may bind to the promoter region of miR-601 were analyzed. It was found that the promoter region of MIR601, host gene of miR-601, contained the binding site of the oncogene c-Myb (Fig. 5a). It has been reported that the nuclear proto-oncogene c-Myb plays a crucial carcinogenic role in the malignance of a variety of cancers^21,22^. Therefore, we hypothesized that c-Myb might regulate the expression of miR-601 at transcriptional level. In order to verify our conjecture, we performed a ChIP assay. The results indicated that c-Myb bound to the promoter of MIR601 (Fig. 5a). Next, three small interfering RNAs targeting c-Myb ware utilized to deplete the expression of c-Myb in OS cells. The efficiency of knockdown was evaluate via conducting RT-qPCR and Western blot experiments (Fig. 5b, c). The luciferase assay suggested depletion of c-Myb significantly promoted the activity of the MIR601 promoter (Fig. 5d). More importantly, c-Myb silencing significantly enhanced the expression of miR-601 in OS cells (Fig. 5e). Additionally, overexpression of c-Myb dramatically repressed the activity of the MIR601 promoter, as well as the expression of miR-601 (Fig. 5f–i).

To further verify the relationship between c-Myb and miR-601, we detected the level of c-Myb in the tissues of OS patients. The results suggested that the level of c-Myb in OS tissues was higher than that in paired normal tissues (Fig. 5j). Correlation analysis results indicated that the expression of c-Myb was negatively correlated with miR-601 in OS tissues (Fig. 5k). Therefore, our results supports that c-Myb is responsible for the inhibition of miR-601 in OS cells.

## Discussion

miRNAs are a class of small, endogenous, evolutionarily highly conserved single-stranded non-coding RNAs that function primarily at the post-transcriptional level of target genes^23,24^. As we all know, the current medical level of the society cannot have a good treatment effect for patients with advanced cancer^25^. Therefore, the study of tumor-related mechanisms is particularly important. With the discovery and understanding of miRNAs and their target gene networks, miRNAs are crucial regulators in the proliferation, apoptosis, cell cycle progression, invasion and metastasis of cancer cells^26^. In this study, miR-601, which has rarely been reported in OS, was selected as the object of study. The results of in vitro and in vivo assay demonstrated that miR-601 plays a crucial role in the proliferation and metastasis of OS as a tumor suppressor. Mechanism exploration have shown that miR-601 is inhibited by c-Myb at the transcriptional level. Meanwhile, miR-601 inhibited the expression of PKMYT1 at the post-transcriptional level, thus exerting its tumor suppressive function.

It has been elucidated that miR-601 is involved in many cancers, including pancreatic cancer, breast cancer, colorectal cancer and so on^27–29^. In these cancers, miR-601 was identified as tumor suppressor. Whereas, the role of miR-601 in OS remains excusive. First of all, through analyzing the data from GEO Datasets, we discovered that miR-601 was reduced in OS cells. The RT-qPCR assay further verified that miR-601 was downregulated in both OS cells and tissues. More importantly, clinical data suggested that OS patients with downregulated miR-601 level had a shorter overall survival. Additionally, a series of functional experiments revealed that high level of miR-601 inhibited the proliferation, invasion and metastasis of OS cells. Therefore, we concluded that miR-601 also functioned as a tumor suppressor in OS.

Studies have demonstrated that PKMYT1 is overexpressed in solid tumors, and elevated PKMYT1 levels are associated with tumor progression, metastasis, and poor prognosis^11,30,31^. In our studies, we found that miR-601 exerted its anti-tumor function via targeting PKMYT1 at post-transcriptional level. Meanwhile, the results indicated that overexpression of PKMYT1 obviously promoted the proliferation, invasion and migration of OS cells. While, the underlying mechanism by which PKMYT1 performs its function in OS needs further verification. According to previous literature, PKMYT1 promoted tumor cell proliferation and apoptosis resistance via activating the MAPK signaling pathway in gastric cancer^32^. In neuroblastoma, PKMYT1 promoted cell growth and inhibited apoptosis through stabilization of the MYCN protein^33^. Meanwhile, PKMYT1 also promotes carcinogenesis of non-small cell lung cancer through the Notch signaling pathway^12^. In hepatocellular carcinoma, PKMYT1 contributes to the enhanced cell growth and motility through the activation of the beta-catenin/TCF signaling^31^. It has also been elucidated that PKMYT1 promote cell proliferation, invasion and migration via targeting sirtuin 3 in ovarian cancer^34^. In addition, studies have shown that PKMYT1 promotes tumor progression of esophageal squamous cell carcinoma by activating AKT signaling pathway^35^. Collectively, PKMYT1 contributes to the malignancy of OS might through a variety of signal pathway.

c-Myb, one of the MYB family members, has been considered as an oncogenic transcription factor involved in various cancers^22,36^. Here we find a potential binding site for c-Myb on the promoter of the miR-601. The ChIP assay results supported that c-Myb could bind to the region of miR-601 promoter. The luciferase assay validated that the activity of miR-601 was enhanced in c-Myb depleted cells and attenuated in c-Myb elevated cells. What’s more, the same trend was observed in the expression of miR-601. The clinical data further supported that miR-601 was negatively related to c-Myb in OS tissues. The above results indicated that c-Myb inhibited the expression of miR-601 via serving as a suppressive transcription factor.

In summary, our results depicted that miR-601 served as an anti-oncogene to suppress cell proliferation and metastasis through inhibition of oncogene PKMYT1. The oncogene c-Myb medicated the inhibition of miR-601 in OS cells. These observations illustrates that miR-601 is a crucial molecule and may provide a novel rationale for the treatment of OS.

## Methods

### Human tissue specimens

All the pairs of normal and OS tissues from a cohort of 66 patients were gotten from Huazhong University of Science and Technology Union Shenzhen Hospital. All OS patients wrote the informed consent. All experiments were approved by medical ethics committee affiliated with Huazhong University of Science and Technology Union Shenzhen Hospital and were carried out in accordance with relevant guidelines and regulations.

### Cell culture

The Cell Bank of China Academy of Sciences (Shanghai, China) provides all the cell lines. α-MEM medium (HyClone, USA) containing 10% FBS (Gibco, USA) was utilized in the use of culturing U2OS, MG63 and MNNG cells. RPMI-1640 medium (Gibco, USA) containing 15% FBS was used to culture Saos-2 cells. DMEM medium (Thermo Fisher Scientific, Waltham, MA, USA) containing 10% FBS was used to culture hFOB1.19 and 143b cells. 100 U/mL penicillin and 100 mg/mL streptomycin were supplemented in all the culture medium. The OS cell lines were cultured under the condition of 37 °C with 5% CO2 in an incubator. hFOB 1.19 cell line was cultured under the condition of 33.5 °C with 5% CO2.

### Western blot

The cells were first lysed with a RIPA lysate and then the concentration of proteins was evaluated via a BCA protein assay kit (Thermo Fisher Scientific). Denaturation of protein by boiling and protein loading buffer. 10% SDS-PAGE was utilized to separate the proteins, which was then transferred to a polyvinylidene fluoride (PVDF, Millipore, Billerica, MA, USA) membranes. After incubated with 5% skimmed milk at room temperature for 1 h, the membranes were incubated with the primary antibodies against c-Myb (1:100, Abcam, ab169111) and PKMYT1 (1:100, Abcam, ab200387) at 4 °C overnight. Then the membranes were incubated with secondary antibody (1:50,000, Abcam, ab6728 or ab6721) conjugated with horseradish peroxidase at room temperature for 1 h. Finally, the Enhanced Chemiluminescence Detection System (Thermo Fisher Scientific) was applied to detect the protein signals. GAPDH served as internal control.

### Cell proliferation assay

Logarithmic phase cells were collected and 2000 cells were added to each well in a 96-well plate with a volume of 100ul. After 1,2,3,4 and 5 days, 20 ul MTT solution (5 mg/ml) was added to each well and continue to be cultured in dark for 4 h. Replaced the liquid in the well with 150 ul dimethyl sulfoxide to each well to dissolve the crystal completely. The absorbance of each well was detected by ELISA reader (Thermo Fisher Scientific) at 490 nm.

### Transwell

The ability of migration and invasion in OS cells was assessed via transwell assay. Place the transwell chambers (8 μm), either uncoated or coated with Matrigel (Sigma), into a 24-well plate. Logarithmic phase cells were collected, diluted with 200ul FBS-free medium, and added into the upper chamber (5 × 10^4^ per well). 700 ul 1640 medium, supplemented with 10% FBS, was added into the lower chamber of the 24-well plate. After 24 h, the liquid in the wells was discarded. Incubate the wells with formaldehyde for 20 min. Subsequently, 0.1% crystal violet was added in to the wells for 15 min, washed with clear water, and gently wiped off the upper cells with cotton swabs. Cells were observed and counted in random 5 fields under the microscope.

### Transfection

The miR-601 mimic, miR-601 inhibitor, short interference RNAs targeting c-Myb and corresponding negative control were purchased from RiboBio (Guangzhou, China). Lipofectamine 2000 (Invitrogen) was utilized for the cell transfection based on the given instructions. Lentivirus vector carrying the miR-601 sequence (LV-miR-601) and negative control (LV-miR-NC) were brought from GeneChem (Shanghai, China). RT-qPCR assay was conducted to evaluate the efficiency of transfection. GAPDH and U6 were served as loading control. The sequence of siRNAs were shown in Table 1.

**Table 1:** Information of the siRNAs and miRNA mimic.

Name	target sequence or (5’–3’)
si–c-Myb-1	AAGCTGAAGAAGCTGGTGGAA
si–c-Myb-2	AAGGACAGCAGACACAGAACC
si–c-Myb-3	CCTCTTAGAATTTGCAGAAAC
NC-siRNA	Sense: UUCUCCGAACGUGUCACGUTT
	Antisense: ACGUGACACGUUCGGAGAATT
miR-601 mimic	GAGGAGGUUGUUAGGAUCUGGU
miR-601 inhibitor	ACCAGAUCCUAACAACCUCCUC
Negative control mimic	UUGUACUACACAAAAGUACUG
Negative control inhibitor	CAGUACUUUUGUGUAGUACAA

### Reverse transcription quantitative polymerase chain reaction (RT-qPCR)

TRIzol Reagent from Invitrogen was applied to extract total RNA from the OS tissues and cultured cells. The cDNA was synthesized via utilizing the PrimeScriptTM RT reagent Kit (Takara, Dalian, China) and miRNA First Strand cDNA Synthesis Kit (Thermo Scientific, Waltham, MA, USA) based on the given instructions. SYBR Premix Ex TaqTM (TaKaRa) was applied to perform the qPCR based on the given protocols. U6 snRNA served as control to normalize the amplified transcript level of miRNA. *β*-actin served as control for the amplified transcript level of mRNA. The primers were shown in Table 2.

**Table 2:** The sequence of PCR primers.

Primer	Sequence
miR-601	Forward: 5’- GAGGAGGTTGTTAGGATCTGGT -3’
	Reverse: Universal PCR Primer R
U6	Forward: Universal U6 Primer F (human)
	Reverse: Universal PCR Primer R
c-Myb	Forward: 5’- GAAGGTCGAACAGGAAGGTTATCT-3’
	Reverse: 5’- GTAACGCTACAGGGTATGGAACA -3’
PKMYT1	Forward: 5’- CATGGCTCCTACGGAGAGGT -3’
	Reverse: 5’- ACATGGAACGCTTTACCGCAT -3’
GAPDH	Forward: 5’- GACTCATGACCACAGTCCATGC -3’
	Reverse: 5’- AGAGGCAGGGATGATGTTCTG -3’

### Luciferase assay

The procedure was same as described previously^37^. To identify the binding between miR-601 and 3’UTR of PKMYT1 mRNA, 3’UTR of PKMYT1 mRNA sequence containing putative binding site of miR-601 (wild type, WT; mutant type, MUT) was constructed into luciferase reporter plasmid. To assess the promoter activity of MIR601, MIR601 promoter sequence containing putative binding site of c-Myb (WT or MUT) was constructed into luciferase reporter plasmid. The U2OS and 143b cells were transfected with luciferase reporter plasmid and pRL-TK plasmid. 48 h later, the luciferase activity was detected via a luciferase activity kit (Promega, USA) according to the directions. Renilla luciferase served as a normalizing control.

### Chromatin immunoprecipitation (ChIP) assay

The procedure was same as described previously^38^. ChIP assay was conducted using a ChIP assay kit (Millipore, New Bedford, MA) according to the given instructions. The cells were grown in a 10 cm petri dish. The formaldehyde was used to crosslink the proteins and DNA. The DNA is interrupted by ultrasound to an average size of 300–500 bp. Take 10 ul of lysate as Input. The lysate was divided into two parts, anti-c-Myb (cell signaling technology, #12,319) and anti-IgG (cell signaling technology, #2729) antibodies were added respectively, and agarose beads were added to precipitate DNA and protein complex. The DNA and protein complex were purified, crosslinking was terminated with 5 M NaCl, and the purified DNA was analyzed by qPCR experiment.

### Biotin miRNA pull-down assay

The single-stranded biotinylated miR-601 or miR-NC probe was brought by the GeneChem (Shanghai, China). Briefly, 4 × 10^6^ U2OS and 143b cells were firstly transfected with 50 μM biotinylated miR-601 or miR-NC for 36 h. Then the cells were lysed followed by the cells lysate were incubated with the biotinylated probe at 4 °C overnight. Then add the streptavidin coated agarose beads (Invitrogen, USA) into the mixture medium that was further incubated at 4 °C for 4 h. Washed and separate the precipitates. Extract total RNA from the precipitates and analyzed the purified RNA via RT-qPCR analysis.

### IHC assay

Firstly, the paraffin sections were dewaxed in xylene and gradient ethanol (100%, 95%, 80%, 70%). After washing with water and PBS, paraffin sections were treated with Triton X-100 and 30% H2O2 for permeability and sealing. After washing again, the slides were placed in a sodium citrate buffer (pH = 6.0, 0.01 M), heated and boiled in the microwave for 10 min. Then 10% goat serum was added for sealing at 37℃ for 30 min. Then the diluted primary antibody against PKMYT1 was added and incubated overnight in the refrigerator at 4 °C. The next day, after washing with PBS, the diluted secondary antibody was added and incubated in an incubator at 37 °C for 1 h. DAB solution was added, and hematoxylin was redyed. Then dehydration was performed, xylene was used to make slices transparent, and neutral resin was added to seal the slices. Finally, the stained sections were imaged at 200X under an inverted microscope.

### In vivo assay

Four-week-old BALB/c nude mice, brought from Shanghai Laboratory Animal Research Center, China, were used for the xenograft model and metastasis model. 2 × 10^6^ LV-miR-601 or LV-miR-NC transfected U2OS cells were subcutaneously injected into the mice (6 per group) in the right hip to construct the xenograft model. Tumor volumes were recorded every 3 days. The mice were sacrificed at 27 days. Solid tumors were removed and weighted. The equation *V* = 0.5 × *L* (length) × *W*^2^ (width) was applied to evaluate the tumor volume. For the metastasis model, 2 × 10^6^ LV-miR-601 or LV-miR-NC transfected U2OS cells were injected into the mice (6 per group) from the tail vein. Lung metastasis was confirmed by H&E staining. The animal experiments were reviewed and approved by the Animal Care and Use Committee affiliated with Huazhong University of Science and Technology Union Shenzhen Hospital and were carried out in accordance with relevant guidelines and regulations in compliance with the ARRIVE guidelines.

## Statistical analysis

All experiments were conducted in triplicate and displayed with one representative experimental result. In addition, the statistics were analyzed by GraphPad Prim version 8.0.0 (GraphPad Software, San Diego, California USA, www.graphpad.com) and SPSS 18.0 software (SPSSlnc., Chicago, IL, USA, www.spss.com). The results are shown as the mean value ± standard deviation. Student’s *t* test and ANOVA were employed to statist the comparisons between groups. Kaplan–Meier survival curves was established with the log-rank test. *P* < 0.05 was considered significant (*P < 0.05; **P < 0.01; ***P < 0.001, NS indicating no significant).

## Supplementary Information

Supplementary Information.
