# Supplementary material for: c-Myb-mediated inhibition of miR-601 in facilitating malignance of osteosarcoma via augmentation of PKMYT1
Source: Sci Rep. 2022 Apr 23;12:6692. doi: 10.1038/s41598-022-10684-0 (PMC9035158; doi:10.1038/s41598-022-10684-0)
Supplement: Supplementary file 1 — Supplementary Information. [file 41598_2022_10684_MOESM1_ESM.pdf]

# Unprocessed original blot/gel images

## Figure 3E

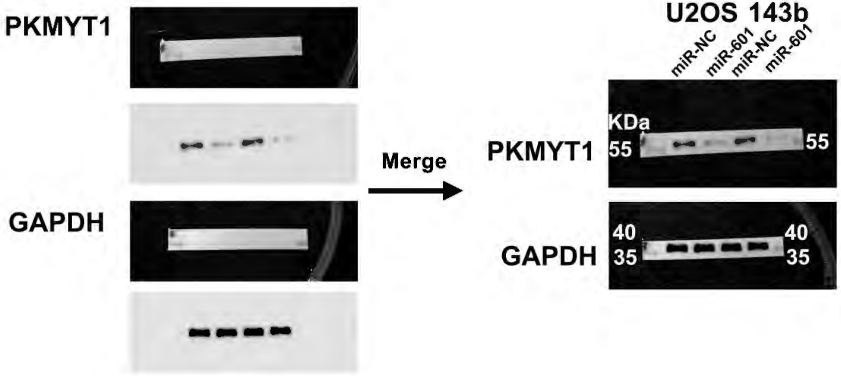


## Figure 3F

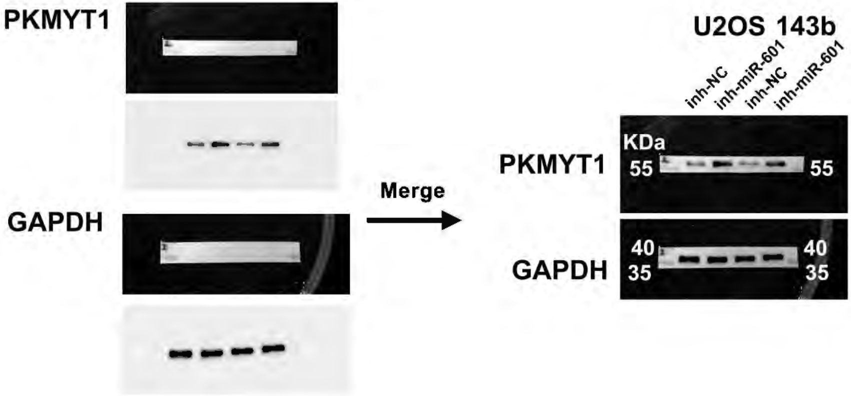

# Unprocessed original blot/gel images

## Figure 4B

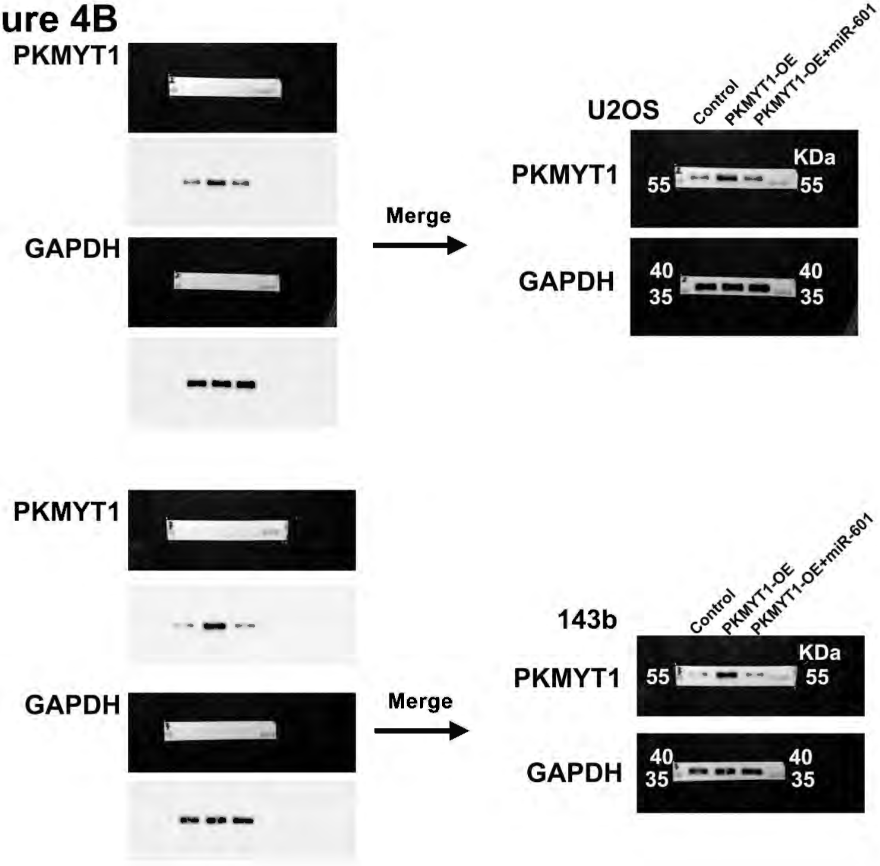

# Unprocessed original blot/gel images

## Figure 5C

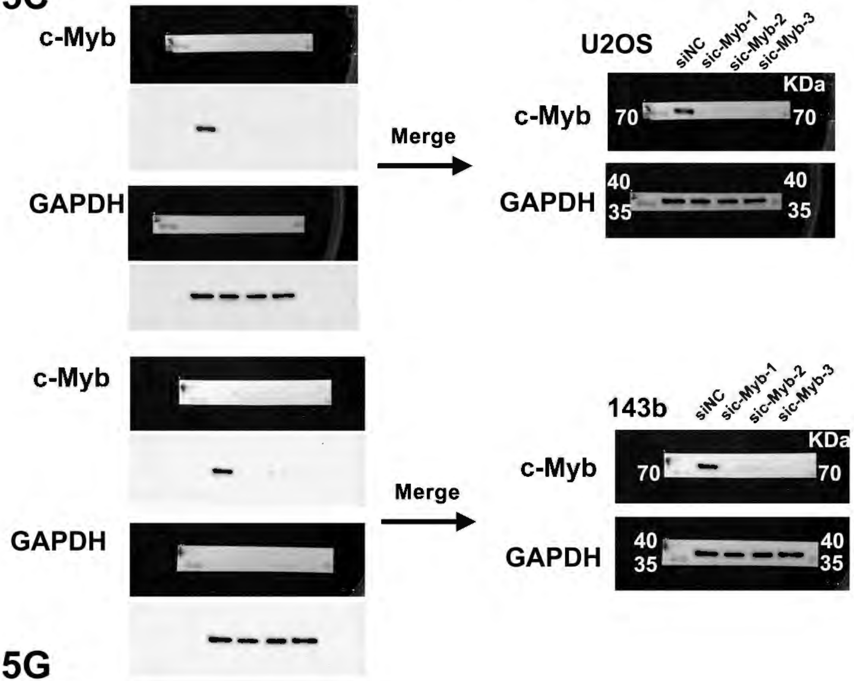


## Figure 5G

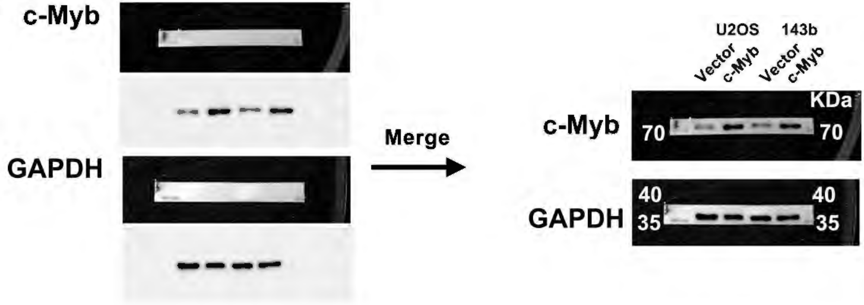

# Certification of no medical ethics

Here is a certification of the article“c-Myb-mediated inhibition of miR-601 in facilitating malignance of osteosarcoma via augmentation of PKMYT1”. The datasets for this article were extracted from the Gene Expression Omnibus (GEO) database. GEO is an open-source database, and will remain publicly available for anyone in the research community to use. The patients involved in the database have obtained ethical approval. The current study does not involve medical ethics, and has been waived from the need for ethics approval.

Author: Peng Luo

Date:2022-03-09
